# SETD2 mutation in renal clear cell carcinoma suppress autophagy via regulation of ATG12

**DOI:** 10.1038/s41419-020-2266-x

**Published:** 2020-01-27

**Authors:** Patricia González-Rodríguez, Pinelopi Engskog-Vlachos, Hanzhao Zhang, Adriana-Natalia Murgoci, Ioannis Zerdes, Bertrand Joseph

**Affiliations:** 1 Institute of Environmental Medicine, Toxicology Unit, Karolinska Institutet, 17177 Stockholm, Sweden; 2 Department of Oncology-Pathology, Karolinska Institutet, 17177 Stockholm, Sweden; 3 Present Address: Department of Neuroscience, Uppsala University, Uppsala, Sweden

**Keywords:** Cancer genetics, Autophagy

## Abstract

Inactivating mutations in the *SETD2* gene, encoding for a nonredundant histone H3 methyltransferase and regulator of transcription, is a frequent molecular feature in clear cell renal cell carcinomas (ccRCC). *SETD2* deficiency is associated with recurrence of ccRCC and bears low prognostic values. Targeting autophagy, a conserved catabolic process with critical functions in maintenance of cellular homeostasis and cell conservation under stress condition, is emerging as a potential therapeutic strategy to combat ccRCC. Epigenetics-based pathways are now appreciated as key components in the regulation of autophagy. However, whether loss of function in the *SETD2* histone modifying enzyme occurring in ccRCC cells may impact on their ability to undergo autophagy remained to be explored. Here, we report that *SETD2* deficiency in RCC cells is associated with the aberrant accumulation of both free ATG12 and of an additional ATG12-containing complex, distinct from the ATG5–ATG12 complex. Rescue of SETD2 functions in the *SETD2* deficiency in RCC cells, or reduction of *SETD2* expression level in RCC cells wild type for this enzyme, demonstrates that SETD2 deficiency in RCC is directly involved in the acquisition of these alterations in the autophagic process. Furthermore, we revealed that deficiency in SETD2, known regulator of alternative splicing, is associated with increased expression of a short ATG12 spliced isoform at the depend of the canonical long ATG12 isoform in RCC cells. The defect in the ATG12-dependent conjugation system was found to be associated with a decrease autophagic flux, in accord with the role for this ubiquitin-like protein conjugation system in autophagosome formation and expansion. Finally, we report that *SETD2* and *ATG12* gene expression levels are associated with favorable respective unfavorable prognosis in ccRCC patients. Collectively, our findings bring further argument for considering the *SETD2* gene status of ccRCC tumors, when therapeutic interventions, such as targeting the autophagic process, are considered to combat these kidney cancers.

## Introduction

Renal cell carcinomas (RCC) are primary malignant adenocarcinomas that originates in the renal tubular epithelium. RCC accounts for around 90–95% of the neoplasms arising from the kidney^1^. Approximately 40% of patients with RCC die because of the disease progression, making of RCC the most lethal and malignant urological tumor. RCC is a heterogeneous group of malignancies with the definition of different histological subtypes, including clear cell renal cell carcinomas (ccRCC), papillary RCC, and chromophobe RCC. However, the ccRCC is predominant, and account for more than 75% of the RCC. The most common feature of ccRCC is the biallelic inactivation of the tumor suppressor gene von Hippel–Lindau (*VHL*) due to chromosome 3p deletion and gene mutation^2,3^. VHL encodes an E3 ubiquitin ligase that targets HIF1α and HIF2α for degradation^4^. VHL loss results in uncontrolled activity of these hypoxia-inducible transcription factors, and the consequent activation of genes involved in the control of angiogenesis, glycolysis, lipogenesis, cell cycle, and apoptosis^5^. Large-scale cancer genomics high-throughput sequencing efforts have brought extra level of molecular complexity to ccRCC with the identification of additional driver genes that might contribute to the disease, beyond the *VHL* gene. Indeed, several genes regulating chromatin remodeling, located on chromosome 3p like *VHL*, including those of the SWI/SNF chromatin remodeling complex (PBRM1 and BAP-1) and the histone modifying enzyme SETD2 are reported to be frequently mutated in ccRCC^6,7^.

De facto, the prevalence of *SETD2*-inactivating mutations in cancer has the highest frequency in ccRCC^8^. *SETD2* mutations are observed in ~10% of human ccRCC primary tumors, and the frequency dramatically increase to ~30% in metastatic ccRCC patient samples, thereby suggesting a role for this genetic alteration in driving the metastatic progression of ccRCC^2,3,7,9^. The loss of SETD2 functions correlates with aggressive clinicophatological features, increased risk of recurrence, and predicts a reduced overall and progression-free survival of ccRCC patients^10–12^. Collectively, these observations argue for a role of *SETD2* inactivation not only in driving the development of tumors, but as well in promoting progression of the disease. SETD2, which stands for Su(var), Enhancer of zeste, Trithorax(SET)-domain containing 2, is a nonredundant methyltransferase responsible for the trimethylation on residues lysine 36 on histone H3 (i.e., H3K36me3) in the gene body of actively transcribed genes^13,14^. SETD2-mediated H3K36me3 promotes transcriptional elongation and plays as well important roles in DNA double-stranded break repair, DNA methylation, and RNA splicing^8^. The loss of SETD2 may therefor cause genomic instability, aberrant transcriptional program, widespread RNA processing defects, and impact on multiple biological processes ranging from cell proliferation, cell differentiation, and cell death^15^.

In the recent years, another biological process, macroautophagy, referred to hereafter as autophagy, has attracted attention in the field of RCC^16^. Autophagy is a catabolic process by which cytoplasmic components are degraded by the lysosome, and is involved in both physiological and pathological conditions^17^. Autophagy comprises a series of dynamic membrane rearrangements orchestrated by a core set of autophagy-related (ATG) proteins^18^. Autophagy involves the assembly of the phagophore, followed by the formation of the autophagosome that contains the cargo to be degraded. Subsequently, autophagosomes fuse with lysosomes, generating autolysosomes, breaking down the cargo by lysosomal enzymes providing energy and macromolecules precursors that can be reused. Although autophagy is primarily a protective process for the cell, it can also contribute to cell death. Hence, interventions to both stimulate and inhibit autophagy have been proposed as cancer therapies^19^. Likewise, inhibition and induction of autophagy have both been considered as therapeutic strategies to combat RCC^20–24^. Additional studies suggest that autophagic gene polymorphisms are associated with ccRCC risk and patient outcome^25,26^. Despite the fact that autophagy is indisputable associated to cytoplasmic events; nuclear events are nowadays considered of importance for this process. Indeed, this process is tightly regulated by epigenetic and associated transcriptional programs, with reported central role for several histone modifying enzymes^27–32^. However, whether the deficiency in the SETD2 histone methyltransferase observed in ccRCC could impact the autophagic core machinery and thereby this biological process is yet to be investigated.

## Results

### SETD2 deficiency in renal cell carcinoma cells is associated with reduced autophagy flux

**Fig. 1:**
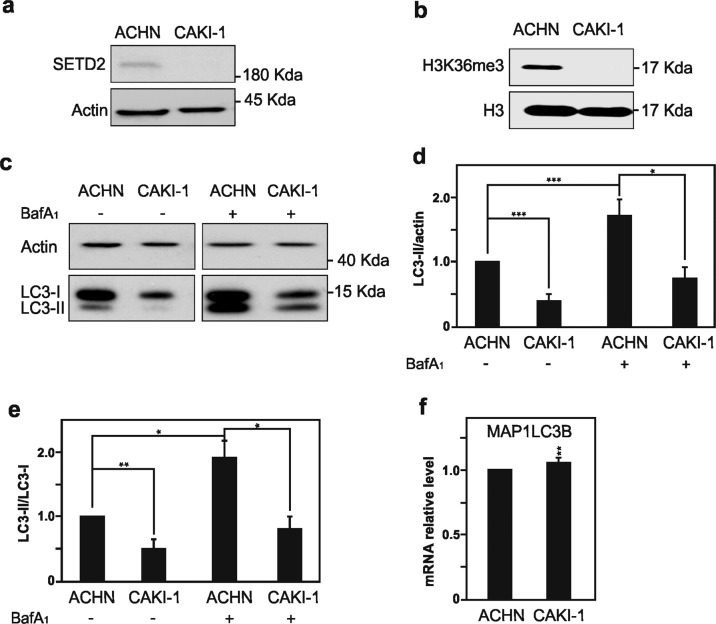
Characterization of SETD2, H3K36me3, LC3-I, and LC3-II expression levels in ACHN and CAKI-1 RCC cells. Immunoblot analysis of the histone modifying enzyme SETD2 (**a**) and its histone target H3K36me3 (**b**) expression levels in ACHN and CAKI-1 renal clear carcinoma cells confirmed the SETD2 deficiency in the last named RCC cell line. **c** Immunoblot analysis of LC3 shows decreased expression level in *SETD2*-deficient CAKI-1 cells as compared with *SETD2-*wild-type ACHN cells. Treatment with Bafilomycin A1 (BafA1, 40 nM) for 4 h shows a significant decrease in LC3-II lipidation in CAKI-1 cells as compared with ACHN cells. **d** Quantification of LC3-II expression compared with the expression of the housekeeping gene, actin, with and without BafA1 treatment in both cell lines. **e** Quantification of LC3-II/LC3-I ratio to monitor autophagic flux in ACHN and CAKI-1 cells with and without BafA1 treatment demonstrates that cells that lacks SETD2 expression exhibit a decreased autophagic flux. **f** Analysis of *MAP1LC3B* mRNA expression by qPCR in *SETD2*-deficient CAKI-1 cells and SETD2-competent ACHN cells. Bars display the mean of four (**d**, **e**) or three (**f**) experiments, error bars represent SEM; ***p ≤ 0.001; **p ≤ 0.01; *p ≤ 0.05.

In order to investigate the impact of SETD2 deficiency could have on the autophagic process in RCC cells, the ACHN cell line, i.e., SETD2-competent RCC cells, and the CAKI-1 cell line, i.e., SETD2-deficient RCC cells were selected (Table 1). Worth a note, these particular RCC cell lines were also selected based on the fact that they are wild type for the *VHL* gene, therefor avoiding one additional gene deficiency, which could impact on the interpretation of the sole effect of SETD2 deficiency. Furthermore, these cell lines are available from cell lines bank depositories and are well characterized in term of their *SETD2* and *VHL* genes mutational status^33,34^. Therefore, first we confirmed by immunoblot analysis the presence of SETD2 protein expression in the ACHN RCC cells, but its absence in the CAKI-1 RCC cells (Fig. 1a). In addition, we checked H3K36me3 expression levels, the histone posttranslational modification that requires the enzymatic activity of SETD2, were also found to be robustly decreased in the SETD2-deficient CAKI-1 cells as compare to the SETD2-competent ACHN cells (Fig. 1b). Next, we monitored the occurrence of baseline autophagy in these RCC cells, as established by an increased lipidation of the main autophagic marker MAP1LC3B, referred as LC3, as it is responsible of substrate recognition to be degraded via autophagy and autophagosome formation. Indeed, occurrence of autophagy results in an increased ratio of the lipidated form (LC3-II) to the unlipidated form (LC3-I), known as LC3 conversion. Our results show that RCC cells with a SETD2 loss of function exhibit a decrease in LC3-II expression level as compared with RCC cells competent for this enzyme when analyzing the ratio between LC3-II/actin (Fig. 1c, d). Since SETD2 is considered to contribute on the regulation of transcription and expression of specific genes, we wondered whether SETD2 lost-of-function promotes the decrease found on LC3 at protein level in RCC. For this purpose, we analyze by reverse transcription polymerase chain reaction (RT-qPCR) the mRNA expression level of *LC3B* gene in both RCC cell lines, which revealed a small significant increase in messenger expression between the *SETD2*-competent and the *SETD2*-deficent cell lines. This data support the idea that the observed decrease in LC3 at protein level in CAKI-1 cells is not the result of a differential transcriptional regulation of *MAP1LC3B* gene in the *SETD2*-deficient RCC cells (Fig. 1f). Moreover, in order to examine whether SETD2 deficiency has an impact on autophagic flux per se, LC3 conversion was assessed in ACHN and CAKI-1 cells in the presence of a late autophagy inhibitor, Bafilomycin A1 (BafA1), which inhibits the fusion of the autophagosome with the lysosome and eventually LC3-II degradation. LC3-II/LC3-I ratio upon BafA1 treatment shows that the CAKI-1 cells that harbor a SETD2 mutation showed a decrease of autophagic flux, when compared with the SETD2-competent AHCN cells (Fig. 1c, e).

**Table 1:** Renal cell carcinoma cell lines, inclusive their *SETD2* and *VHL* status, used in this study.

	Research resource identifier	Cell line collections	Disease	*VHL* status; *SETD2* status (COSMIC) + for wild-type gene − for mutated gene	References
**ACHN**	RRID:CVCL_1067	ATCC® CRL-1611™; ECACC 88100508	Renal cell carcinoma, derived from metastatic site, male, 22Y	*VHL*+; *SETD2*+	^33,55,56^
**Caki-1**	RRID:CVCL_0234	ATCC® HTB-46™; DSMZ ACC-731	Renal cell carcinoma, derived from metastatic site, male, 49Y	*VHL*+; ***SETD2***−	^33,56,57^
**Caki-2**	RRID:CVCL_0235	ATCC® HTB-47™; DSMZ ACC-54; ECACC 93120819	Renal cell carcinoma, derived from primary tumor site, male, 69Y	***VHL***−; *SETD2*+	^33,57^
**A498**	RRID:CVCL_1056	ATCC® HTB-44™; DSMZ ACC-55	Renal cell carcinoma, derived from primary tumor site, male, 52Y	***VHL*−**; ***SETD2***−	^33,56,57^
**RCC-FG2**	RRID:CVCL_5873	CLS #300249	Renal cell carcinoma, derived from primary tumor site, male, 77Y	***VHL***−; ***SETD2*−**	^58,59^
**769-P**	RRID:CVCL_1050	ATCC® CRL-1933™; CLS #300106	Renal cell carcinoma, derived from primary tumor site, female, 63Y	***VHL***−; *SETD2*+	^60,61^

*ATCC* American Type Culture Collection, *DZME* German Collection of Microorganisms and Cell Culture; *ECACC* European Collection of Authenticated Cell Cultures, *CLS* Cell Line Service, *COSMIC* Catalog of Somatic Mutations in Cancer

**Fig. 2:**
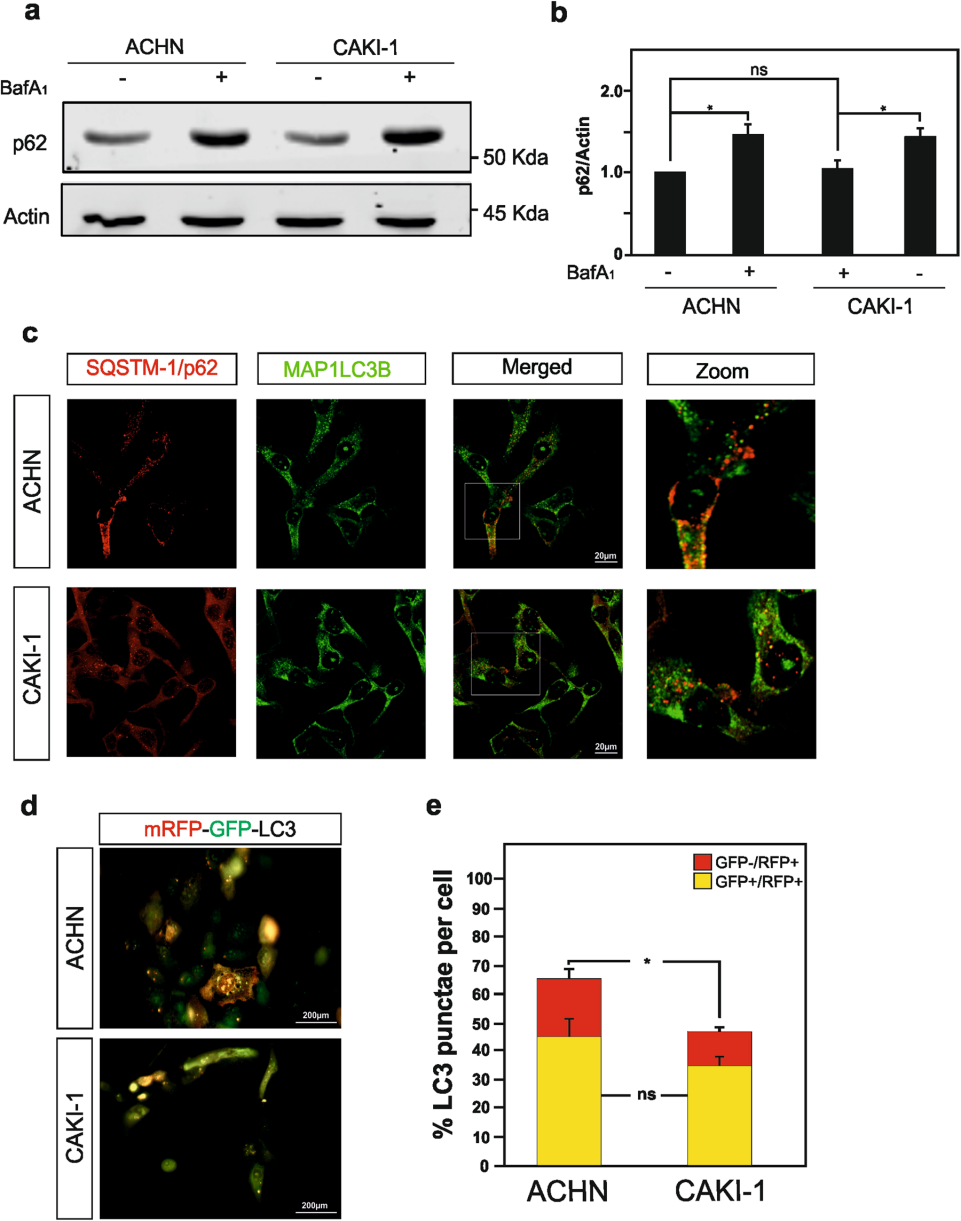
RCC cells deficient for SETD2 expression shows a decrease in autophagic flux. **a** Immunoblot analysis, and its quantification in **b**, of SQSTM1/p62 expression level in *SETD2*-deficient CAKI-1 cells and SETD2-competent ACHN cells show a modest increase in p62 protein upon BafA1 treatment (but not in untreated conditions) in the CAKI-1 cells as compared with ACHN cells. **c** Immunofluorescence analysis of SQSTM1/p62 and MAP1LC3B reveals decreased colocalization of these two proteins in the *SETD2*-deficient RCC cells. **d**, **e** Both RCC cell lines were transfected with the tandem reporter construct, mRFP-GFP-LC3 that allows distinguishing between autophagosomes (GFP+/RFP+ yellow puncta) and autolysosomes (GFP−/RFP+ red puncta). Representative immunofluorescence images for mRFP-GFP-LC3 transfected ACHN and CAKI-1 cells are depicted in **d**. **e** Quantification of GFP+/RFP+ yellow puncta and GFP−/RFP+ red puncta in at least 30 cells per experimental condition shows a significant decrease on autolysosomes number. Bars display the mean of three experiments, error bars represent SEM; ns nonsignificant; **p ≤ 0.01.

The ubiquitin-associated protein p62 (also known as Sequestosome-1), an adapter protein, which possesses a short-LC3 interaction region that facilitates its direct interaction with LC3, is also used to monitor autophagy^35,36^. p62 is an autophagosome cargo protein that targets other proteins that bind to it for selective degradation via autophagy. Degradation of p62 is another widely used readout to monitor autophagic activity as p62 directly binds to LC3 and is selectively degraded by autophagy. Therefore, cells undergoing autophagy should demonstrate the colocalization of p62 and LC3B. Immunoblot analysis reveals that p62 protein is found to be expressed at similar level in ACHN and CAKI-1 RCC cells, and that p62 accumulates in CAKI-1 cells when autophagy was inhibited using BafA1 treatment (Fig. 2a, b). Moreover, immunofluorescence microscopy imaging for both LC3B and p62 proteins exposed a significant decrease in autophagosome formation in CAKI-1 cells, as compared with ACHN cells, as illustrated by the reduced LC3B/p62 colocalization (Fig. 2c).

To further confirm the impact of SETD2 deficiency on the autophagic flux in RCC cells, we took advantage of a tandem reporter construct, mRFP-GFP-LC3^37^. The green fluorescence of this tandem reporter is attenuated in the acidic pH lysosomal environment, whereas the mRFP is not. Therefore, the green fluorescent component of the composite yellow fluorescence from this mRFP-GFP-LC3 reporter is lost upon autophagosome fusion with a lysosome, whereas the red fluorescence remains detectable. Thus, this probe allows distinction between autophagosomes (GFP+/RFP+ yellow puncta) and autolysosomes (GFP−/RFP+ red puncta). Our results obtained show that as expected CAKI-1 cells present an overall lower number of LC3 punctae cells, as well as significantly less red-color-labeled LC3 puncta, i.e., autolysosomes. In addition, SETD2-dedicient CAKI-1 cells show a reduced autophagic flux in the SETD2-deficient cells, as compared with the SETD2-competent cells, were further confirm by the mRFP-GFP-LC3 autophagic flux assay (Fig. 2d, e).

Collectively, these data indicate a reduced autophagic flux in the SETD2-deficient CAKI-1 RCC cells, which is not due to a transcriptional decrease of *LC3* expression, as compared with the ACHN RCC cells, which carry a wild-type version of this methyltransferase enzyme.

### ATG12 complexes and free ATG12 accumulate in SETD2-deficient renal cell carcinoma cells

**Fig. 3:**
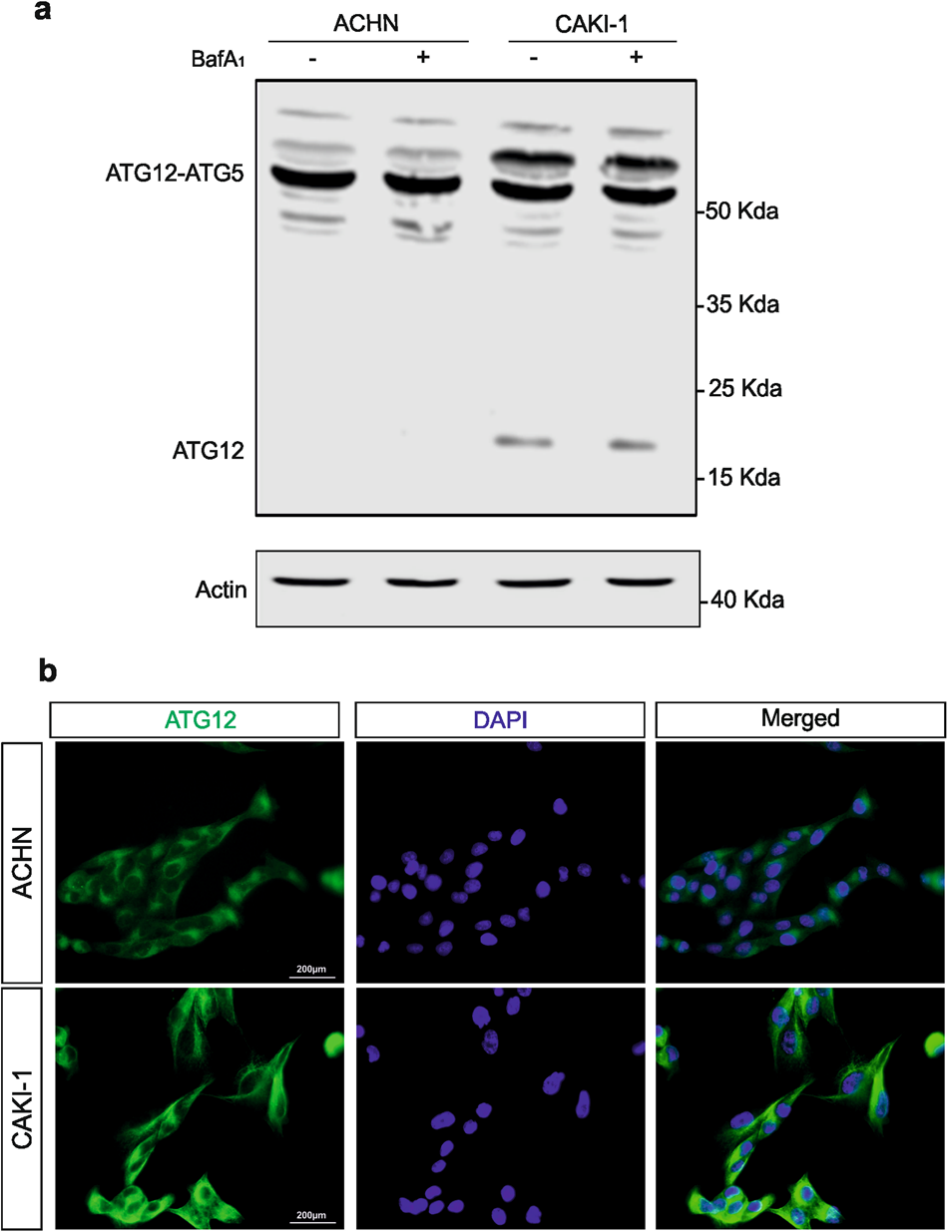
Free ATG12 and ATG12-associated complexes expression is increased in SETD2-deficient cells. **a** Immunoblot analysis of ATG12 protein in *SETD2*-deficient CAKI-1 cells and *SETD2*-competent ACHN cells, reveal the accumulation of both free ATG12 and of an additional ATG12-containing complex, distinct from the ATG5–ATG12 complex, in the *SETD2*-deficient RCC cells. **b** Immunofluorescence analysis shows an increase expression for ATG12 in CAKI-1 cells as compared with ACHN cells.

Thereafter, in order to get insights on how SETD2 deficiency could impact on the autophagic flux, we undertaken to look at key component of the autophagy core machinery that contribute to the control of autophagosome formation and expansion. Autophagy relies on the activities of two ubiquitin-like protein conjugation systems, which catalyze the covalent conjugation of ATG12–ATG5 and therefore addiction of the lipid phosphatidylethanolamine to LC3. A common E1-like enzyme, ATG7, facilitates these reactions. Thereafter, ATG12–ATG5 conjugate proceeds to form an active multimeric complex together with autophagy-related 16-like 1 (ATG16L1) that localizes to sites of autophagosome assembly and promote the conjugation of phosphatidylethanolamine to LC3B^38,39^. In fact, deficiencies in the *Atg5, Atg7*, and *Atg12* core autophagy genes have all been reported to impair induced and constitutive autophagy in mouse models^40–42^. Hence, ATG7 expression, as well as the formation of the ATG5–ATG12 covalent complexes were investigated by immunobloting in ACHN and CAKI-1 RCC cells. No major differences in ATG7 expression level were observed in SETD2-deficient versus SETD2-competent RCC cells (Supplementary Fig. 1a, b). However, striking differences were noted when ATG12 immunoblot was performed. Indeed, this experimental set revealed that in the CAKI-1 cells, i.e., SETD2-deficient RCC cells, exhibit a higher ATG12 expression level, and detection of immunoreactive bands, in addition to the one corresponding to the ATG5–ATG12 covalent complex, for free ATG12 protein, as well as for an additional ATG12-containing complex around 60 kDa. In contrast, ACHN cells, SETD2-competent RCC cells, mainly exhibited the ATG12-immunoreactive band corresponding to the ATG5–ATG12 conjugate but not free ATG12 (Fig. 3a). Immunofluorescence imaging for ATG12 protein confirmed its higher expression level, in punctate structure, in CAKI-1 cells as compared with ACHN cells (Fig. 3b).

**Fig. 4:**
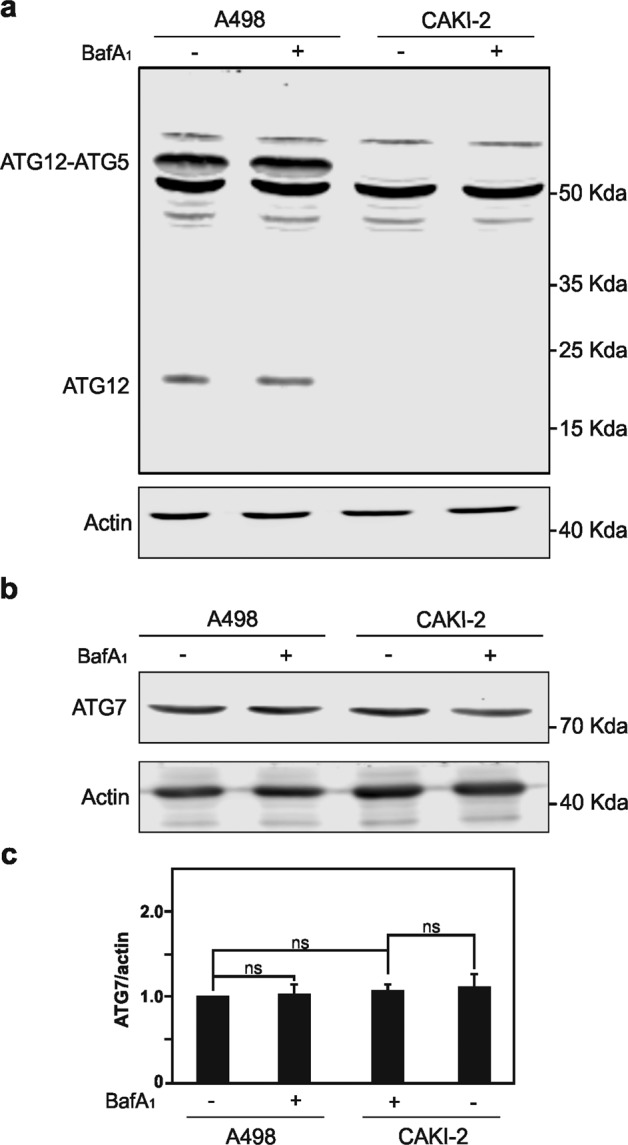
*VHL* mutation in A498 and CAKI-2 RCC cells does not impact on the accumulation of free ATG12, and ATG12 forming complexes. **a** Immunoblot analysis of ATG12 protein in CAKI-2 characterized by *VHL* gene mutation but a wild-type *SETD2* gene, and A498 characterized by both *VHL* and *SETD2* (loss of function) gene mutation revealed that the accumulation in free ATG12, and the ATG12-containing complex, distinct from the ATG5–ATG12 complex, is independent of the *VHL* gene status. **b** Immunoblot analysis, and its quantification in **c**, of ATG7 expression does not show striking differences between those two RCC cell lines. Bars represent the mean of three independent experiments, error bars represent SEM. Ns nonsignificant.

Moreover, to assess whether the accumulation of free ATG12 and the additional ATG12-containing complex is SETD2 dependent, four additional RCC cell lines, CAKI-2 and 769-P both lines characterized by *VHL* gene mutation but a wild-type *SETD2* gene, as well as A498 and RCC-FG2 both lines characterized by *VHL* and *SETD2* (loss of function) gene mutation were analyzed (Table 1). Immunoblot analysis for SETD2 protein expression, as well as H3K36me3 expression levels for these additional RCC cell lines, which confirm the SETD2 loss of function in A498 and RCC-FG2 RCC cells are depicted in Supplementary Figs. 2a, b and 3a, b. We also explored whether *VHL* gene mutation, the most prevalent reported gene deficiency in RCC cells, could impact on the accumulation of free ATG12 and occurrence of ATG12 additional complex. Immunoblot analysis demonstrated that the aberrant accumulation of free ATG12 protein and of the additional ATG12-containing complex can be observed in all RCC cells deficient for SETD2, i.e., A498, Caki-1, and RCC-FG2 RCC cells, and that the VHL status of the cells did not impact in the acquisition of these characteristics suggesting that the phenotype observed is clearly due to SETD2 deficiency and VHL does not play role on ATG12 regulation (Fig. 4a and Supplementary Fig. 3c, d). Of note, the VHL status of the RCC cells did not either impact on ATG7 expression levels. (Fig. 4b, c). Immunofluorescence imaging for ATG12 protein indicated higher expression level in RCC-FG2 RCC cells as compared with 769-P RCC cells (Supplementary Fig. 3e). A reduced autophagic flux, as illustrated by reduced LC3-II/LC3-I ratio upon BafA1 treatment, in the SETD2-deficient A498 and RCC-FG2 RCC cells, as compared with the CAKI-2 and 769-P RCC cells, which carry a wild-type version of this methyltransferase enzyme was also observed (Supplementary Figs. 2c, d and 3f, g).

### Occurrence of free ATG12 and aberrant ATG12-containing complex in renal cell carcinoma cells is SETD2 dependent

Hence, we report that SETD2 deficiency in RCC cells is associated with the aberrant accumulation of both free ATG12 and of an additional ATG12-containing complex, distinct from the ATG5–ATG12 complex, which also appears to be associated with reduced autophagic flux in these cells. In order to gain evidence that SETD2 deficiency in RCC is directly involved in the acquisition of these defects in the autophagic process, experiments aiming at the gain and loss of function of SETD2 in RCC cells were undertaken.

**Fig. 5:**
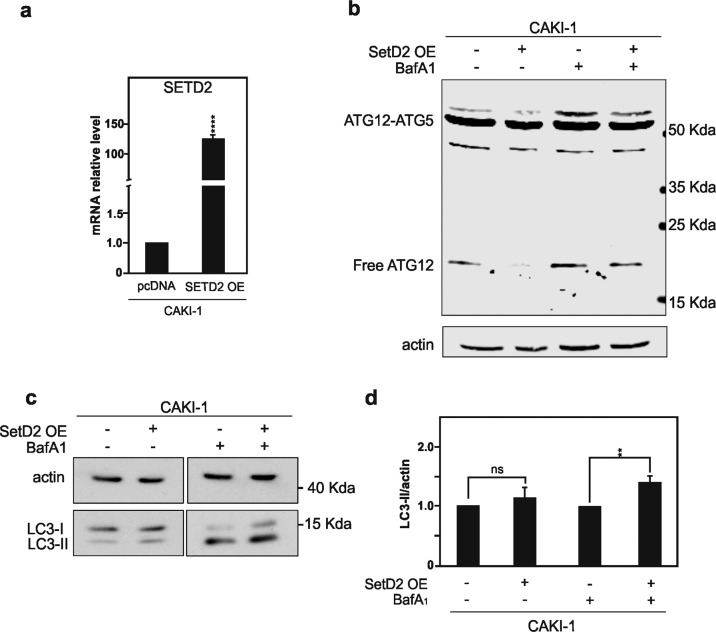
Rescue of SETD2 expression in *SETD2*-deficient CAKI-1 cells lead to decreased expression of free ATG12 and ATG12-containing complexes and increased of LC3-II lipidation. **a**–**d** *SETD2*-deficient CAKI-1 cells were transfected with an expression vector encoding for SETD2. **a** qPCR analysis of *SETD2* mRNA expression level in *SETD2*-deficient CAKI-1 cells upon *SETD2* overexpression. **b** Immunoblot analysis of ATG12 protein reveals that rescue of SETD2 expression in CAKI-1 lead to decrease in observed levels of free ATG12, and the ATG12-containing complex, distinct from the ATG5–ATG12 complex. **c** Immunoblot analysis, and its quantification in **d**, of LC3B expression upon those conditions shows increase LC3-II lipidation in CAKI-1 cells. Bars represent the mean of three independent experiments, error bars represent SEM. Ns nonsignificant; *p ≤ 0.05; ****p ≤ 0.0001.

**Fig. 6:**
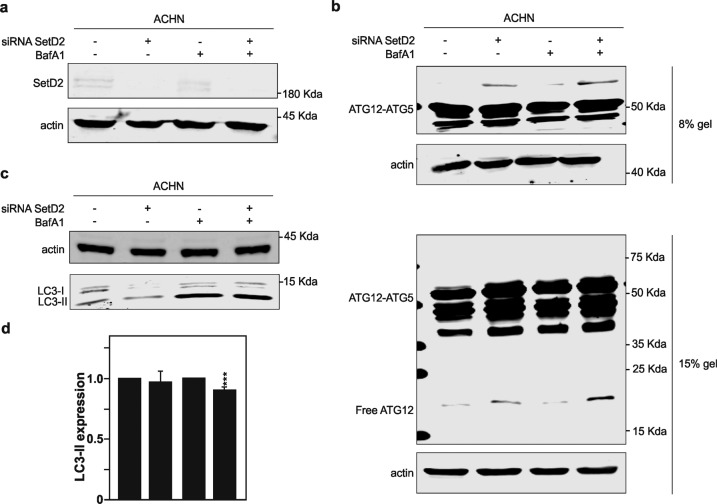
Downregulation of SETD2 expression in SETD2-competent ACHN cells lead to increase of an accumulation of free ATG12 and ATG12 complexes and decrease in LC3-II lipidation. **a**–**d** Downregulation of SETD2 expression in the *SETD2*-competent ACHN cells was achieved by small-interfering RNA targeting *SETD2*. **a** Immunoblot analysis for SETD2 confirm the reduction in SETD2 expression in ACHN cells upon treatment with a siRNA directed against *SETD2* for 48 h. **b** Immunoblot analysis of ATG12 protein reveals that the downregulation of SETD2 expression in the ACHN *SETD2*-competent cells promotes the increase in free ATG12 and ATG12-associated complexes. **c** Analysis of LC3B expression in upon downregulation of SETD2 expression. Bafilomycin A1 (40 nm) was used for 4 h treatment. **d** Quantification of LC3-II levels normalized to actin. Bars represent the analysis of three independent experiments, error bars represent SEM; ***p ≤ 0.001.

Rescue of SETD2 functions in the CAKI-1 cells was achieved by the transient overexpression of an expression vector encoding for the enzyme as shown by RT-qPCR (Fig. 5a). Remarkably, reintroducing wild-type SETD2 in CAKI-1 that are otherwise deficient for the enzyme was enough to significantly reduce the presence of both free ATG12 and of the additional ATG12-containing complex in these cells, as shown by immunoblotting analysis (Fig. 5b). Similar reductions were also observed in CAKI-1 cells overexpressing SETD2 and treated with the late inhibitor of autophagy BafA1. On the other hand, complementary experiments were performed in which *SETD2* gene expression was targeted using a pool of four small-interfering RNAs (siRNAs) in the ACHN cells, wild type for the enzyme. As expected, the *SETD2* siRNAs pool leads to robust downregulation of SETD2 protein expression level in ACHN RCC cells (Fig. 6a). Decrease SETD2 protein expression on its own was able to promote the appearance of both free ATG12 and of the additional ATG12-containing complex in ACHN RCC cells (Fig. 6b). Blocking the autophagic flux with BafA1 treatment further increase the accumulation of these ATG12-immunoreactive bands in the immunoblots.

### SETD2 deficiency in renal cell carcinoma cells is associated with increase expression of a ATG12 short spliced isoform

Thereafter, we wanted to elucidate how SETD2 deficiency in RCC cells could have an impact on the expression of ATG12, and potentially control to the expression of different ATG12 variants that could lead to occurrence of free ATG12 and additional ATG12-containing complexes. SETD2-mediated H3K36 trimethylation has been implicated in the regulation of alternative splicing^43–45^. In fact, posttranslational modification of histone tails is closely associated with the regulation of this process and H3K36me3 is of particular interest as the levels of this specific histone mark differ based on exon utilization, with alternatively spliced exons having lower levels of H3K36me3 than those that are constitutively included^44,46^. Furthermore, altering *SETD2* gene expression levels is enough to influence the inclusion of exons in genes known to be alternatively spliced^43^. Finally and of importance for the current investigation, H3K36me3-deficient ccRCC tumors have been reported to show alterations in splicing and evidence of intron retention^15^.

**Fig. 7:**
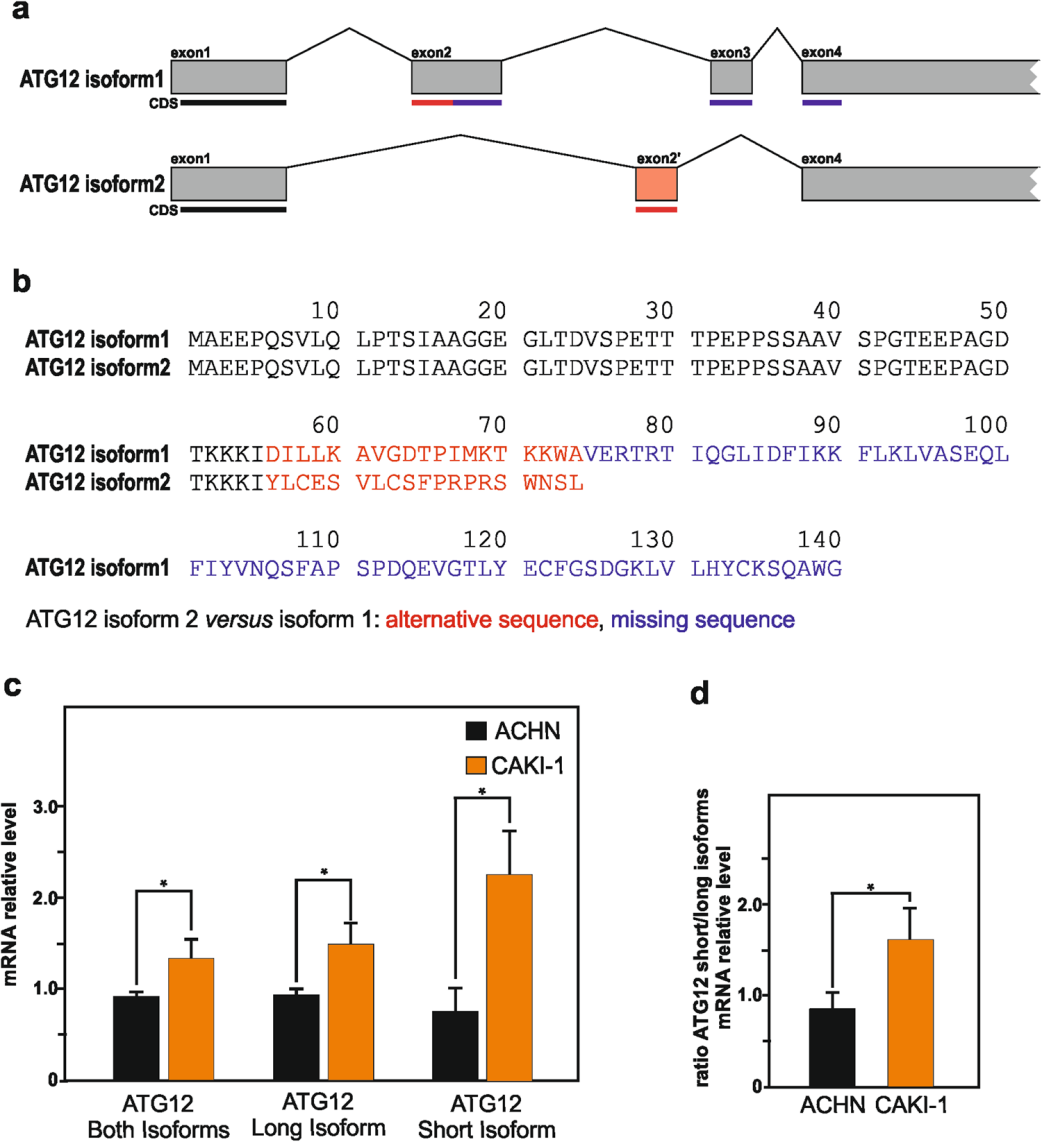
SETD2 deficiency in RCC cells is associated with increase expression of a ATG12 short spliced isoform. **a** Illustration of *ATG12* long isoform 1 and *ATG12* short isoform exons and introns structures, as well as indication of the location of the coding sequences (CDS) in the exons. **b** Amino acid sequences for ATG12 long isoform 1 (with UniProt identifier O94817-1) and ATG12 short isoform 2 (with UniProt identifier O94817-4). For the two isoforms, shared amino acid sequences are reported in black color, alternative sequences are reported in red color, whereas the missing sequence in the short isoform is reported in blue color in the long isoform. **c**, **d** qPCR analysis of mRNA expression level for ATG12 short and long isoforms, or both of them, in *SETD2*-deficient CAKI-1 cells and SETD2-competent ACHN cells revealed significantly higher expression (**c**), as well as increased ratio between ATG12 short isoform versus ATG12 long isoform (**d**) in SETD2-deficient RCC cells. Bars represent the analysis of three independent experiments, error bars represent SEM; *p ≤ 0.05.

In fact, UniProt, database of protein sequence and functional information available at uniport.org, provide information about the human ATG12 protein (accession number O94817). This entry describes the existence of two isoforms, with respective length of 140 and 74 amino acid residues, suggested to be generated by alternative splicing. The isoform 1, the longest one, with identifier O94817-1 is considered as the canonical sequence. The shortest isoform 2 with identifier O94817-4, which lacks an exon in the coding region compared with the isoform 1, present a shorter and distinct carboxyl-terminus. Whereas the transcript for ATG12 isoform 1 mRNA originate from four distinct exons, the transcript for the shortest ATG12 isoform, i.e., isoform 2, only originate from three exons (Fig. 7a). De facto, the sequence for this variant differs from the canonical sequence as follows: an alternative sequence for residues 56–74 (DILLKAVGDTPIMKTKKWA → YLCESVLCSFPRPRSWNSL), and the sequence for residues 75–140 is missing (Fig. 7b).

Given the strong evidence for SETD2-dependent regulation of alternative splicing, and the suggested existence of ATG12 isoforms issue of alternative splicing for, we speculated that dysregulation of SETD2 might impact on the differential expression of these ATG12 isoforms in RCC cells. Taking advantage of the unique sequences present in their carboxyl-terminus of each ATG12 isoform, we selected primers allowing the individual quantification of their messenger expression by qPCR (Supplementary Fig. 4). This mRNA analysis revealed that SETD2-deficient RCC cells exhibited a significantly higher expression of the ATG12 short isoform as compared with RCC cells carrying a wild-type version of SETD2. The overall ATG12 messenger expression, as well as the ratio between ATG12 short isoform versus ATG12 long isoform were also found to be increased in the SETD2-deficient RCC cells (Fig. 7c, d). Collectively, these data indicate that deficiency in SETD2, known regulator of alternative splicing, is associated with increased expression of the ATG12 short isoform to the depend of the canonical ATG12 long isoform in RCC cells.

### Manipulation of ATG12 expression in renal cell carcinoma cells impact on their autophagy and cell migration capability

Thereafter, since we could establish that SETD2 status in RCC cells is a key determinant for the aberrant presence of free ATG12 and additional ATG12-containing complex in these cells, we decided to investigate how SETD2 manipulation in these cells could affect the autophagic flux per se. The SETD2 rescue in CAKI-1 not only impacted on the presence of the additional ATG12-immunoreactive bands in immunoblots, but as well on the autophaghic flux as demonstrated by the increased LC3 conversion even observed in the presence of BafA1 treatment (Fig. 5c, d). When the autophagic flux was tested in ACHN cells, looking at LC3 conversion, it appears that SETD2 downregulation using siRNA directed against *SETD2* leads to a significant decrease in LC3-II levels, which was more obvious in the cells treated with BafA1 (Fig. 7c, d).

Finally, with the purpose of exploring whether the above described deficiency in autophagy could impact on other cellular properties of RCC cells, we undertook to analysis whether the manipulation of SETD2 could impact on the migration capabilities of these cells. Indeed, autophagy has recently been described as a regulator of cell migration^48,49^. Confluent RCC cell monolayers were subjected to a wound-healing assay to monitor cell motility. ACHN, SETD2-competent cells, were transfected with siRNAs targeting SETD2 or as control scramble siRNAs, whereas CAKI-1, SETD2-deficient cells, were transfected with an expression vector encoding for SETD2, or an empty expression vector as control, 24 h before wounding. Confluent cell cultures were scraped with a pipet tip to create a cell-free wound and images were captured at the beginning and at regular intervals during cell migration to close the wound.

A significant increase in wound-healing (cell motility) activity was seen in ACHN cells in which the expression of SETD2 had been targeted by an antisense approach velocity, as compared with the ACHN cells control, transfected with a scramble siRNA (Supplementary Fig. 5a, b). Quite the opposite, CAKI-1 cells in which the expression of SETD2 had been restored by overexpression exhibited a substantial decrease in the migration rate (Supplementary Fig. 5c, d). Thus, these data establish that SETD2 expression in RCC cells impact on their have cell migration capability.

### In clear cell RCC patients SETD2 and ATG12 gene expression levels are associated with favorable respective unfavorable prognosis

**Fig. 8:**
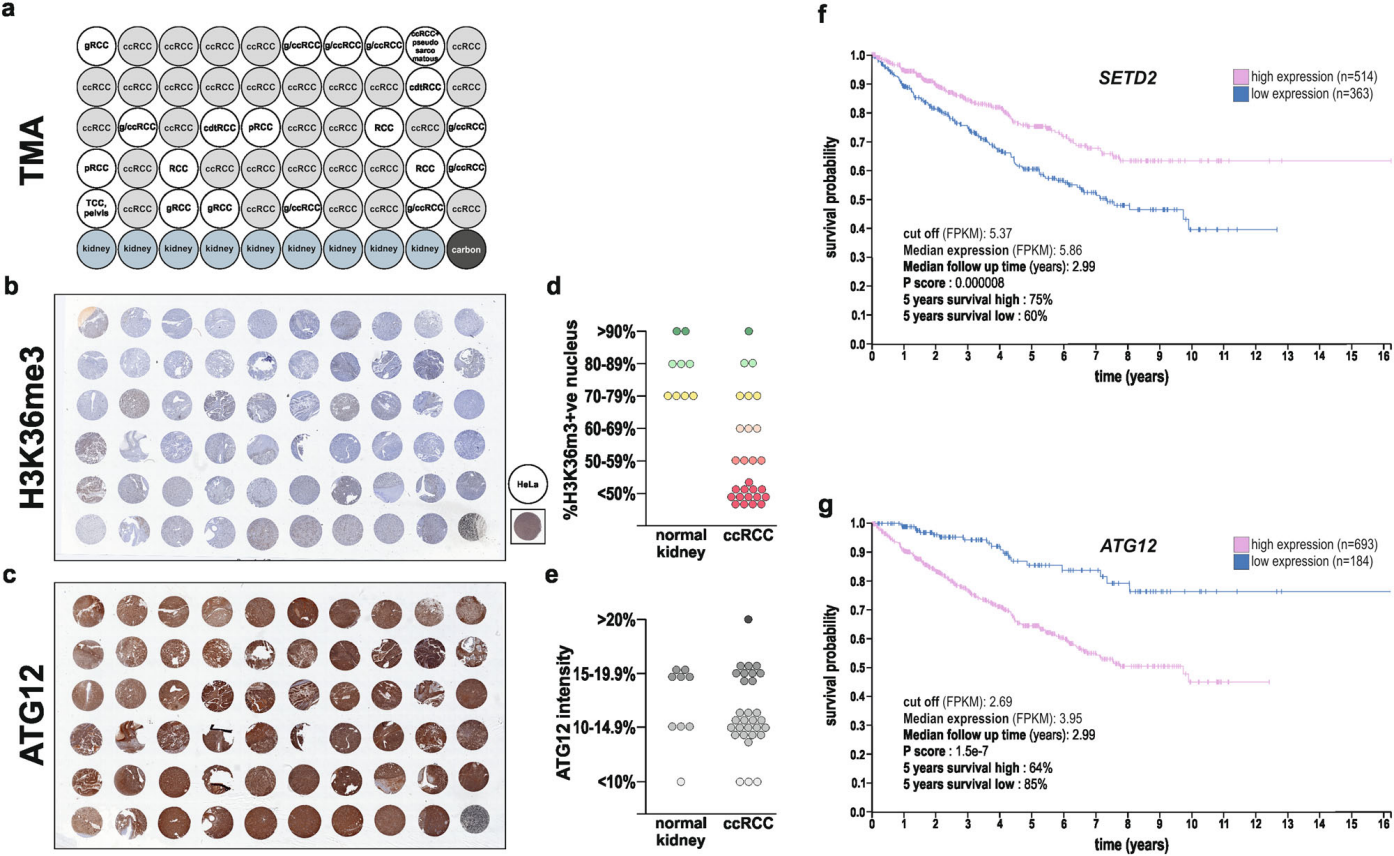
Low H3K36me3, but high ATG12 protein expression levels are observed in ccRCC tumors, and *SETD2* and *ATG12* gene expression levels have prognostic values for ccRCC patients. **a** Human kidney tissue microarrays including 30 tissues classified as ccRCC tumors (highlighted in light gray) were proceed for immunohistochemical staining for H3K36me3 (**b**) and ATG12 protein (**c**) and revealed low H3K36me3 (**d**) but high ATG12 (**e**) protein expressions. HeLa were used as a positive control for H3K36me3 protein expression in **b**. **f**, **g** *SETD2* and *ATG12* high versus low gene expression levels (expressed in FPKM and with a cut off of 5.37 for *SETD2* and 4.66 for *ATG12*) in tumor tissue derived from ccRCC patients at the time of diagnosis and their correlation to patient survival, i.e., follow up after diagnosis (expressed in years) was extracted from the pathology atlas of the human cancer transcriptome available at the Human Protein Atlas (www.proteinatlas.org). Survival analysis, prognosis of each group of patients examined using Kaplan–Meier survival estimators, and survival outcomes of the groups compared by log-rank tests revealed that *SETD2* should be considered as a favorable prognostic gene and *ATG12* as an unfavorable prognostic gene for ccRCC patients.

Since we observe that SETD2 deficiency is associated with the appearance of free ATG12, as well as the expression additional ATG12-containing complexes, and an overall increase in total ATG12 protein expression levels, we decided to investigate whether *ATG12* gene expression levels, as well as the expression of the gene, i.e., *SETD2*, which product is causing the global increase in ATG12 expression levels, could be used as survival prognostic factors for ccRCC patients. First, we took advantage of a human kidney TMA that include 30 ccRCC tumors (Fig. 8a). Immunohistochemistry analysis of H3K36me3 expression levels, used as a readout for SETD2 enzymatic activity, as well as ATG12 protein expression, confirmed low H3K36me3, but high ATG12 expression levels in most of the ccRCC tumors biopsies (Fig. 8b–e and Supplementary Fig. 6a–c). Thereafter, we looked at the available gene expression data sets from the pathology atlas of the human cancer transcriptome^47^ available at the Human Protein Atlas to explore whether the expression levels of the *SETD2* and *ATG12* genes could both be associated with a survival prognosis for patients suffering from ccRCC. Based on the Fragments Per Kilobase of transcript, per Million mapped reads (FPKM) value of each gene, ccRCC patients were classified into two expression groups, high and low expression (a FPKM of 5.37 and 4.66 were used as cut off for *SETD2* and *ATG12* expression, respectively) and the correlation between expression level and patient survival was examined. The prognosis of each group of patients was examined by the Human Protein Atlas using Kaplan–Meier survival estimators, and the survival outcomes of the two groups were compared by log-rank tests. Supporting our findings, the analysis of these gene expression data set revealed that *SETD2* should be considered as a favorable prognostic gene whereas *ATG12* as an unfavorable prognostic gene for ccRCC patients (Fig. 8f, g).

## Discussion

In recent years, *SETD2* has attracted interest as a potential tumor suppressor gene, whose inactivation would therefor participate to tumor initiation and progression for a wide range of human tumors, including epithelial, central nervous system, and hematopoietic tumors^6,50–53^. However, SETD2-inactivating mutations distinguish themselves in the ccRCC cancer, where they are most prevalent than in any other cancer type^8,50^. Furthermore, these mutations correlate with aggressive clinicophatological features, and are associated with an unfavorable prognosis in patients with ccRCC^10–12^. Thus, given its frequency of inactivation in ccRCC, its critical role as a tumor suppressor, and its possible use as progression marker in ccRCC, effort have been placed to elucidate the biological consequences of SETD2 loss of function in ccRCC cells. Here, we report that SETD2-inactivating mutations have a significant impact on the autophagy. Mechanistically, we uncovered a SETD2 loss of function-dependent occurrence of an aberrant ATG12-containing complex, in addition to the conventional ATG5/ATG12 covalent complex, as well as increased of free ATG12, in RCC cells carrying the *SETD2* gene mutation. Furthermore, we confirmed the existence of two distinct ATG12 isoforms that had been suggested in protein and gene databases, a canonical long isoform and a short isoform generated by alternative splicing, and revealed that SETD2 deficiency in RCC cells promote a significant increase in the expression levels of the short isoform. Whereas, the presence of the short ATG12 isoform in SETD2-deficient cells is responsible for the presence of additional ATG12-containing complex and free ATG12, which appears based on its molecular weight to be the long ATG12 isoform, potentially by competing it will require further investigations. The apparent defect in the ATG12-dependent conjugation system was associated with decrease autophagic flux, in accordance with the role for this ubiquitin-like protein conjugation system in autophagosome formation and expansion^38,39^. Considering the impact autophagy can have on cancer cells survival or dismiss^54^, targeting the autophagy process has been considered as potential therapeutic strategy to combat various type of tumors, including in patient with ccRCC^20–24^.

Previous studies reported that polymorphisms in autophagic gene are associated with ccRCC risk and patient outcome, indicating deficiencies in the autophagic core machinery impact in ccRCC patients^25,26^. Our investigation, bring an additional level of understanding, with the discovery that the prevalent *SETD2* gene mutation in ccRCC, directly impact on this autophagic core machinery and thereby reduce the autophagic flux. We also expose that *SETD2*, as well as *ATG12* gene expression, can present prognostic value for ccRCC patients, where low *SETD2* expression but high *ATG12* gene expressions are associated with an unfavorable prognosis. Thus, we argue that the *SETD2* gene status of ccRCC tumors should be taken into account when therapeutic intervention aiming at the autophagic process is undertaken. This consideration could contribute to better prognosis of such interventions, including in the clinics, and provide more personalized cancer therapy for ccRCC patients.

## Material and methods

### Cell culture

ACHN, CAKI-1, CAKI-2, RCC-FG2, A498, and 769-P human ccRCC cell lines^33,55–61^, described in Table 1, were cultured in Dulbecco’s modified Eagle’s Medium (DMEM) with Glutamax®, supplemented with 10% fetal bovine serum and 1% with Penicillin/Streptomycin (Gibco). The cells were grown in 75cm^2^-flasks under standard conditions at 37 °C and 5% CO_2_.

### Reagents and antibodies

Bafilomycin A1 was purchased from Santa Cruz Biotechnology (sc-201550). Information about antibodies used in this study are provided in Table 2.

**Table 2:** Antibody used in this study.

	Research resource identifier	Application	Sources
**β-actin** (mouse mAb)	RRID:AB_262137	Western blot	Sigma Aldrich (A-3853)
**ATG7** (rabbit mAb)	RRID:AB_10625656	Western blot	Genetex (GTX61647)
**ATG12** (rabbit pAb)	RRID:AB_10703973	Western blot, IF	Abcam (ab155589)
**G3PDH** (rabbit pAb)	RRID:AB_2107456	Western blot	Trevigen (2275-PC-100)
**H3K36me3** (rabbit pAb)	RRID:AB_1950412	Western blot	Cell Signaling (4909)
**LC3B** (rabbit pAb)	RRID:AB_796155	Western blot	Sigma Aldrich (L7543)
**SETD2** (rabbit pAb)	RRID:AB_2811237	Western blot	Genetex (GTX127905)
**SQSTM1/p62** (mouse mAb)	RRID:AB_945626	Western blot, IF	Abcam (ab56416)

### Transfection of ACHN RCC cells with SETD2 siRNA

ACHN human RCC cells were seeded in six-well-plate dishes and transfected 24 h after platting either with nontargeting siRNA (used as control) or siRNA against *SETD2* (50 nM) with 6 µL of lipofectamine 3000 (Invitrogen), respectively. *SETD2* (L-012448-00) with the following four validated siRNAs: UAAAGGAGGUAUAUCGAAU, GAGAGGUACUCGAUCAUAA, GCUCAGAGUUAACGUUUGA, and CCAAAGAUUCAGACAUAUA and nontargeting ON-TARGET (D-001810) SMARTpool siRNAs with the following four validated siRNAs: UGGUUUACAUGUCGACUAA, UGGUUUACAUGUUGUGUGA, UGGUUUACAUGUUUUCUGA, and UGGUUUACAUGUUUUCCUA, were purchased from Dharmacon. The cells were treated when indicated, harvested and analyzed 48 h after transfection.

### SETD2 overexpression in SETD2-deficient RCC cells

CAKI-1 and A498 RCC cells were seeded in six-well plates. The following day, cells were transfected with 2 µg of SETD2 plasmid or pcDNA3.1, respectively, with lipofectamine 3000 by following the manufacturer’s protocol. 24 h after transfection, cells were subjected to the corresponding analysis.

### mRFP-GFP-LC3 assay

Cells were grown on coverslips in six-well dishes and after 24 h were transfected with lipofectamine 3000 (Invitrogen) and 2 µg of the mRFP-GFP-LC3 plasmid per well. After 24 h transfection, cells were fixed by incubation in 4% paraformaldehyde for 7 min at room temperature followed by nuclei staining using in situ mounting medium with DAPI (Duolink®). Autophagy flux was determined counting the number of LC3 positive cells. The mRFP-GFP-LC3 plasmid was a kind gift of Dr Tamotsu Yoshimori (Osaka University, Japan)^37^. Samples were analyzed with Axio Zoom V16, Stemi 305, Zeiss Microscope.

### Western blot analysis

Cells were seeded in six-well plates and transfected the day after (see above). The cells were harvested by using a cell scraper and lysed with Laemmli buffer (62.5 mM Tris-HCl pH 6.8, 2% SDS, 10% Glycerol, 5% β-mercaptoethanol, and 0.02% Bromophenol Blue). Obtained samples were sonicated (Bioruptor® Pico, Diagenode) and boiled 8 min at 99 °C. Whole-cell lysate was resolved by SDS-PAGE gels (8 or 15% acrylamide) and transferred onto nitrocellulose membranes (0.2 or 0.45 µm-pores) using wet transfer (Bio-Rad). The membranes were blocked with 5% milk in PBS-Tween 20 0.1% for 1 h and incubated overnight at 4 °C with the corresponding primary antibody. Furthermore, followed by incubation with the appropriate IRDye® Secondary Antibody (LI-COR, 1:10,000) for 1 h at room temperature 20–25 °C. Immunoblot with anti-β-actin or G3PDH antibody was used for standardization of protein loading. Details about antibodies used in this study can be found in Table 2. Bands were visualized using an Odyssey CLx infrared imaging system (LI-COR Bioscience). All targeted proteins of interest were normalized to the selected housekeeping gene, intensity of the bands were verified within the same linear range and quantification was performed using the ImageJ software.

### Immunofluorescence

ccRCC cells were seeded in six-well plates. The following day cells were washed twice with PBS 1× and fix with 4% paraformaldehyde for 15 min at room temperature. Cells were then incubated with blocking/permeabilization (B/P) buffer (10 mM HEPES, 0.3% Tx-100, 3% BSA) for 1 h at room temperature, following with the corresponding antibodies diluted in B/P buffer overnight at 4 °C. The following day, the samples were washed three times for 5 min in PBS 1× and incubated with the secondary antibody in second B/P buffer (0.2% Tween 20, 3% BSA) for 1 h at room temperature. Cells were washed two times with 1× PBS and mounted with in situ mounting medium with DAPI (Duolink®). Secondary antibodies, Alexa Fluor® 488 Goat anti-Rabbit IgGs (1:500) and Alexa Fluor® Goat anti-Mouse IgGs (1:500) (Invitrogen), were used. Samples were analyzed with Axio Zoom V16, Stemi 305, Zeiss Microscope.

### Quantitative real time PCR

ACHN and CAKI-1 cells were seeded in six-well plates and harvested after 24 h. Total RNA isolation was performed following the manufacturer’s instructions of RNeasy mini kit (Qiagen). RNA quantifications of the different samples were determined using a NanoDrop® spectrophotometer (Thermo Fisher Scientific). cDNA was synthesized from 1 µg of RNA using Oligo (dT) primers, dNTP mix, and superscript IV (Invitrogen). qPCR was run on an ABI 7500 (Applied Biosystems) using SYBR™ Green Master Mix (Qiagen). *GAPDH* was used as a housekeeping gene. QPCR statistical analysis was performed using R. Primers used:

MAP1LC3B_rev: 5′-CTGTAAGCGCCTTCTAATTATC-3′;

MAP1LC3B_fwd: 5′-ATAGAACGATACAAGGGTGAG-3′;

SETD2_rev: 5′- CTCCTTTAGGTCTTTCCAAC-3′;

SETD2_fdw: 5′-AGAACAGCCAGATAAAACAG-3′;

GAPDH_rev: 5′-TTTTTGGTTGAGCACAGG-3′;

GAPDH_fwd: 5′-ACAGTTGCCATGTAGACC-3′.

Location and sequence of the primers used for quantification of the ATG12 long and short isoform, or both of them are indicated in Supplementary Fig. 4.

### Tissue microarray analysis

The human kidney tissue microarray (TMA) consisted of 59 cores of patients with renal tumors among those 9 cores correspond to normal tissue adjacent to the cancer. The TMA (Novus Biologicals; NBP2-30220) consists of tissue sections of 4 μm thickness, prepared from the TMA formalin-fixed paraffin-embedded tissue blocks and immunostaining was performed as following. First, slides were dried for 1 h in an oven at 60°. For deparaffinization process, slides were submerged in xylene for 20 min, rehydration using descanting ethanol concentrations (100–95–70%) to water (5 min, two times) and antigen retrieval for 20 min under microwave treatment. The AR9 buffer (1:10 dilution in peroxidase-free water; Perkin Elmer, Boston, MA, USA) was used as the unmasking buffer, while sections were then cooled in washing buffer (PBS-Tween 20, 0.1%). Peroxide and protein block were then performed for 10 and 5 min respectively with ready-to-use reagents (Ultravision LP Detection System HRP DAB, ThermoScientific, Waltham, MA, USA) before the application of primary antibody. Primary antibodies used were directed against Tri-Methyl-Histone H3 (Lys36) (1:100; (D5A7) XP® Rabbit monoclonal antibody; Cell signaling (#4909)) and ATG12 (1:100; Rabbit polyclonal antibody, Abcam (ab155589)) and left overnight at 4°. The day after, primary antibody enhancer and secondary detection HRP-Polymer reagents applied were ready to use (Ultravision LP Detection System HRP DAB, ThermoScientific, Waltham, MA, USA) and were combined with DAB chromogenic substrate (ThermoScientific, Waltham, MA, USA). Counterstaining was performed using hematoxylin, sections were de-hydrated with ascending concentrations of ethanol (70–95–100%-xylene) and slides were coversliped using a permanent mounting media (Pertex, Histolab, Gothenburg, Sweden).

TMA slides were digitally scanned at an original magnification of 20× using Glissando High-Performance Desktop Scanner (Objective Imaging Ltd, Cambridge, UK) and immunohistochemical expression scoring was performed using ImageJ software v. 1.48 (NIH, Bethedsa, MD, USA) by two independent investigators. For the tri-methyl-histone H3 (Lys36) antibody expression, the number of cells with nuclear marker positivity was estimated and their percentage out of the total cell number per TMA was calculated. For the ATG12 expression, the median intensity score (in pixels) was measured for every TMA core. Healthy/normal renal tissue was served as an internal control of IHC expression for both antibodies. HeLa cells were used as a positive control for tri-methyl-histone H3 (Lys36) antibody expression.

### Survival analysis

*SETD2* and *ATG12* gene expression levels (expressed in FPKM) in the tumor tissue at the time of diagnosis and their correlation to patient survival, i.e., follow up after diagnosis (expressed in years) were extracted from the pathology atlas of the human cancer transcriptome available at the Human Protein Atlas (www.proteinatlas.org)^47^. Raw data related to *SETD2* and *ATG12* for ccRCC tumors are available at the following URLs:

*SETD2:* proteinatlas.org/ENSG00000145782-ATG12/pathology/renal+cancer/KIRC.

*ATG12:* proteinatlas.org/ENSG00000181555-SETD2/pathology/renal+cancer/KIRC.

### Statistical analysis

For the in vitro analysis, statistical analysis was performed using GraphPad Prism (GraphPad Software, Version 6.0), the threshold for statistical significance was considered when the p-value was equal or less than 0.05. For the survival analysis, the prognosis of each group of patients was examined by the Human Protein Atlas using Kaplan–Meier survival estimators, and the survival outcomes of the two groups were compared by log-rank tests.

## Supplementary information

Supplementary figure legends

Supplementary figure 1

Supplementary figure 2

Supplementary figure 3

Supplementary figure 4

Supplementary figure 5

Supplementary figure 6
