# Supplementary material for: SETD2 mutation in renal clear cell carcinoma suppress autophagy via regulation of ATG12
Source: Cell Death Dis. 2020 Jan 27;11(1):69. doi: 10.1038/s41419-020-2266-x (PMC6985262; doi:10.1038/s41419-020-2266-x)
Supplement: Supplementary file 1 — Supplementary figure legends [file 41419_2020_2266_MOESM1_ESM.docx]

**Supplementary Figure Legends**

**Supplementary Figure 1 | ATG7 is expressed at similar levels in RCC cells independently of their *SETD2* status.**

**a** Immunoblot analysis, and its quantification in panel **b**, of ATG7 expression in *SETD2*-deficient CAKI-1 cells and *SETD2*-competent ACHN cells, treated or not with BafA1, shows no differences in protein expression.

**Supplementary Figure 2 | Characterization of SETD2, H3K36me3, LC3I and LC3II expression levels in CAKI-2 and A498 RCC cells.**

**a, b** Immunoblot analysis of the histone modifying enzyme SETD2 (**a**) and its histone target H3K36me3 (**b**) expression levels in CAKI-2 and A498 cells confirmed the SETD2 loss of function in the last named RCC cell line. **c** Immunoblot analysis of LC3 and **d** quantification of LC3-II/LC3-I ratio to monitor autophagic flux in CAKI-2 and A498 cells with and without BafA1 treatment demonstrates that cells that lacks SETD2 expression exhibit a decreased autophagic flux.

Bars display the mean of three experiments, error bars represent SEM; ns non-significant; * p≤0.05; ** p≤0.01.

**Supplementary Figure 3 | Characterization of SETD2, H3K36me, free ATG12 and ATG12 associated complexes as well as autophagic flux in RCC-FG2 and 769-P RCC cells.**

**a, b** Immunoblot analysis of the histone modifying enzyme SETD2 (**a**) and its histone target H3K36me3 (**b**) expression levels in 769-P and RCC-FG2 cells confirmed the SETD2 loss of function in the last named RCC cell line. **c, d** Immunoblot analysis of ATG12 protein in *SETD2*-deficient RCC-FG2 cells and *SETD2*-competent 769-P cells, reveal the accumulation of both free ATG12 and of an additional ATG12-containing complex, distinct from the ATG5-ATG12 complex, in the *SETD2*-deficient RCC cells. **e** Immunofluorescence analysis shows an increase expression for ATG12 in RCC-FG2 cells as compared to 769-P cells. **f** Immunoblot analysis of LC3 and **g** quantification of LC3-II/LC3-I ratio to monitor autophagic flux in RCC-FG2 and 769-P cells with and without BafA1 treatment demonstrates that cells that lacks SETD2 expression exhibit a decreased autophagic flux.

Bars represent the analysis of three independent experiments, error bars represent SEM; * p≤0.05; ** p≤0.01.

**Supplementary Figure 4 | *ATG12* long isoform 1 and *ATG12* short isoform 2 coding regions, corresponding amino acid sequences, and primers used to analysis their mRNA expression.**

Nucleic acid sequences and corresponding amino acid sequences for the two ATG12 isoforms, *i.e.* ATG12 long isoform 1 and ATG12 short isoform 2, are depicted. Location of the primers pairs used to amplify the long isoform 1, short isoform 2 or both of them are indicated in the nucleic acid sequences. Start codons as well as stop codons are boxed. The nucleic acid sequence cover the exon 1 shared by both isoforms (highlighted in dark gray), exon 2 and 3 for isoform 1 (highlighted in light and dark gray, respectively), the alternative spliced exon 2’ for isoform 2 (highlighted in orange), and part of the exon 4 shared by both isoforms (highlighted in light gray).

**Supplementary Figure 5 | SETD2-deficiency contributes to increased migration capability of RCC cells.**

**a** to **d** Confluent RCC cell monolayers were subjected to a wound-healing assay to monitor cell motility. ACHN, SETD2-competent cells, were transfected with a siRNA targeting SETD2 or as control a scramble siRNA (**a, b**), whereas CAKI-1, SETD2-deficient cells, were transfected with an expression vector encoding for SETD2, or an empty expression vector as control (**c, d**), 24 hours before wounding. **a** Whereas a significant decrease in cell motility was observed in CAKI-1 cells in which the expression of SETD2 had been restored by overexpression (quantification in panel **b**). **c** ACHN cells in which the expression of SETD2 had been targeted by an antisense approach, exhibited a significant increase in velocity as compared to the ACHN cells control, transfected with a scramble siRNA (quantification in panel **d**).

**Supplementary Figure 6 | Tissue microarray analysis.**

**a** human kidney tissue microarrays including 30 tissues classified as ccRCC tumors (highlighted in light grey), were proceed for immunohistochemical staining for H3K36me3 and ATG12 protein as depicted in figure 8. **b** For the tri-methyl-histone H3 (Lys36) antibody expression, the number of cells with nuclear marker positivity was estimated and their percentage out of the total cell number per TMA was calculated. **c** For the ATG12 expression, the median intensity score (in pixels) was measured for every TMA core.
